# Seasonal Variation in the Spatial Distribution of Basking Sharks (*Cetorhinus maximus*) in the Lower Bay of Fundy, Canada

**DOI:** 10.1371/journal.pone.0082074

**Published:** 2013-12-04

**Authors:** Zachary A. Siders, Andrew J. Westgate, David W. Johnston, Laurie D. Murison, Heather N. Koopman

**Affiliations:** 1 Fisheries and Aquatic Sciences, University of Florida, Gainesville, Florida, United States of America; 2 Grand Manan Whale and Seabird Research Station, Grand Manan, New Brunswick, Canada; 3 Department of Biology and Marine Biology, University of North Carolina Wilmington, Wilmington, North Carolina, United States of America; 4 Nicholas School of the Environment, Duke University, Beaufort, North Carolina, United States of America; Università degli Studi di Napoli Federico II, Italy

## Abstract

The local distribution of basking sharks in the Bay of Fundy (BoF) is unknown despite frequent occurrences in the area from May to November. Defining this species’ spatial habitat use is critical for accurately assessing its *Special Concern* conservation status in Atlantic Canada. We developed maximum entropy distribution models for the lower BoF and the northeast Gulf of Maine (GoM) to describe spatiotemporal variation in habitat use of basking sharks. Under the Maxent framework, we assessed model responses and distribution shifts in relation to known migratory behavior and local prey dynamics. We used 10 years (2002-2011) of basking shark surface sightings from July-October acquired during boat-based surveys in relation to chlorophyll-*a* concentration, sea surface temperature, bathymetric features, and distance to seafloor contours to assess habitat suitability. Maximum entropy estimations were selected based on AICc criterion and used to predict habitat utilizing three model-fitting routines as well as converted to binary suitable/non-suitable habitat using the maximum sensitivity and specificity threshold. All models predicted habitat better than random (AUC values >0.796). From July-September, a majority of habitat was in the BoF, in waters >100 m deep, and in the Grand Manan Basin. In October, a majority of the habitat shifted southward into the GoM and to areas >200 m deep. Model responses suggest that suitable habitat from July - October is dependent on a mix of distance to the 0, 100, 150, and 200 m contours but in some models on sea surface temperature (July) and chlorophyll-*a* (August and September). Our results reveal temporally dynamic habitat use of basking sharks within the BoF and GoM. The relative importance of predictor variables suggests that prey dynamics constrained the species distribution in the BoF. Also, suitable habitat shifted minimally from July-September providing opportunities to conserve the species during peak abundance in the region.

## Introduction

Basking sharks (*Cetorhinus maximus*, Gunnerus 1765) are obligate filter feeders of zooplankton, primarily copepods, and are found circumglobally in temperate and tropical seas [1]. Despite being the second largest fish in the world, surprisingly little is known about their ecology [2]. Basking sharks have been subjected to high levels of anthropogenic mortality and this, combined with low reproductive rates, threatens many populations [3–5]. Basking sharks are listed globally as *Vulnerable* [6] and of S*pecial Concern* in Atlantic Canada [7]. Moreover, the species is listed as *Endangered* in the North Pacific and eastern North Atlantic (eNA), regions, which historically supported large populations [8,9]. Consequently, the future recovery of basking sharks may depend on protecting remaining areas of high shark density [10,11]. 

Considerable seasonal changes in the regional distributions of this species [12–15] complicate conservation efforts [16]. In eNA, basking sharks exhibit spatial distribution shifts, as exemplified by the seasonal migration from the neritic zone in the summer to the offshore continental shelf in the winter [2, 16]). Considerably less is known about their distribution in the western North Atlantic (wNA) where animals are rarely observed outside of inshore coastal waters in the summer [12,17–19]. Within the Gulf of Maine (GoM), Owen (1984) observed shifts in basking shark sighting records from offshore areas during the spring to inshore areas in the summer and subsequent disappearance in the fall. In stark contrast to the eNA, Skomal et al. (2009) tracked basking sharks from Massachusetts in the summer to as far south as Brazil in the winter. 

Basking sharks occur throughout the wNA, but to date their principal habitats have not been identified [19]. One suspected region is the Bay of Fundy (BoF), located between New Brunswick and Nova Scotia, Canada [19]. The BoF experiences an extreme tidal range (8 -16 m) and a seasonal cyclonic gyre near its mouth [20], which promotes zooplankton retention [21] and increased densities of foraging planktivores [22–25]. For example, high densities of North Atlantic (NA) right whales (*Eubalaena glacialis*), co-occur in the BoF where the dominant zooplankton (energy-rich *Calanus finmarchicus* stage V [C5] copepodites) aggregate and comprise over 79% of the zooplankton biomass [26–28]. Similarly, basking shark sightings data positively correlate with the greatest C5 abundance (Murison, L.D., unpublished data) suggesting these megaplanktivores are also targeting this energy-dense prey source [29]. 

Species distribution models offer a method to predict important habitat based on sighting records and to relate environmental variables to species occurrence [30,31]. The relatively new but robust method of maximum entropy distribution modeling (Maxent) makes use of presence-only datasets [32,33], which include archival, citizen science, and unstandardized survey records. This model routine has been used recently to investigate and describe the distributions of several marine megafauna [34–40], including North Atlantic right whales [41]. 

To quantify the distribution of basking sharks in the lower BoF we applied Maxent analysis to surface sighting records collected between 2002 to 2011 from scientific boat surveys and commercial whale-watching vessels. We investigated how basking shark distribution changed seasonally (July-October) and responded to abiotic and biotic factors. We also present several new considerations regarding the application of this technique to marine data and discuss these over two frameworks: 1) discriminating basking shark habitat; and 2) variation due to model-fitting in large datasets. 

## Materials and Methods

### Presence-only sighting data

**Figure 1:**
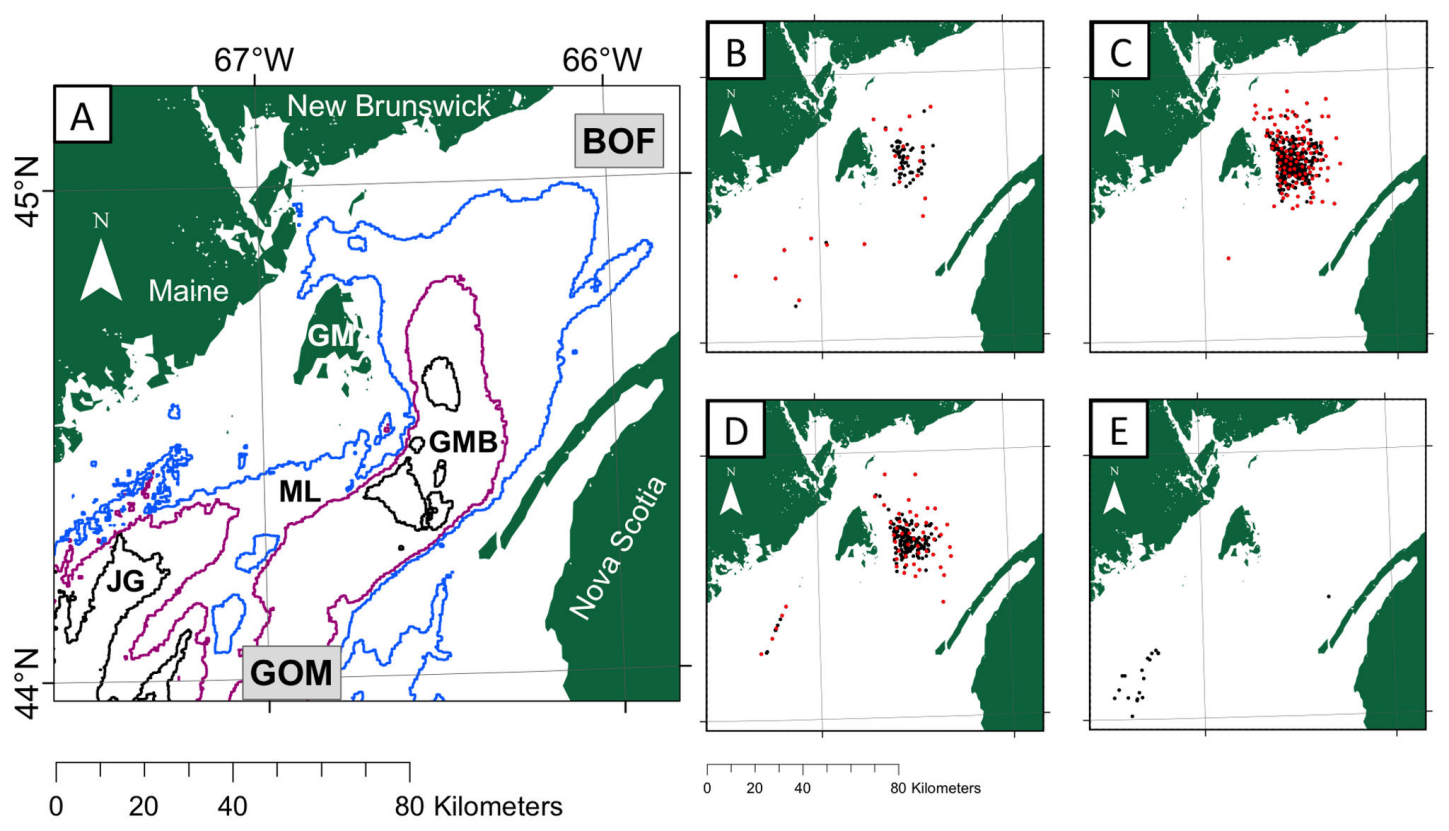
Study area and sightings data of basking sharks in the Bay of Fundy. (A) Map of the study area in the lower Bay of Fundy (BOF) and northeastern Gulf of Maine (GOM) with 100 m (blue), 150 m (purple), and 200 m (black) contour lines depicted. GM refers to Grand Manan Island, JG to Jones Ground, ML to Murr Ledges, GMB to the Grand Manan Basin. Maps depicting the sightings data (black) and the sightings left after the average nearest neighbor decimation (red) for July (B), August (C), September (D), and October (E). Sightings were collected by a commercial whale-watch organization and New England Aquarium [NEAq] from 2002 to 2011, and were used to generate *Cetorhinus* *maximus* distribution models.

Basking shark surface sightings were obtained from two long-term (2002-2011) datasets. The first was from non-standardized surveys made during commercial whale-watching trips conducted from North Head, Grand Manan (Figure 1A). The second consisted of sightings collected incidentally during surveys for NA right whales [42] conducted by the New England Aquarium (NEAq) [43]. These independent data sets encompassed 10 years and covered an area of 7539 km^2^ in the lower BoF and northeastern GoM between 44° - 45°N and 66° - 67° W (Figure 1A). We collated sightings irrespective of the data source and included all animals independently to represent the number of sharks present at that location. A total of 884 sharks were sighted: 90 in July (10.18% of all sightings), 554 in August (62.67%), 221 in September (25%), and 19 in October (2.15%). Basking sharks were sighted throughout the study area in July through October but the large majority of sightings occurred off the eastern side of Grand Manan in the Grand Manan Basin (Figure 1 A, B, & D). For August, the entirety of sightings was located within the Bay of Fundy off the eastern side of Grand Manan (Figure 1 C). In October, the majority of sightings (18 out of 19) were sighted in Jones Ground in the Gulf of Maine (Figure 1E). We assumed that the locations of surface sightings mirror the locations of suitable underwater habitat. However, basking sharks move horizontally underwater, so data from basking shark diving profiles (274 dives/833 hours; Siders, unpublished data) was employed to validate this assumption. From these profiles, average horizontal distance was found by multiplying the mean vertical movement rate for descents (-0.22 m s^-1^) and ascents (0.22 m s^-1^) by the mean descent (189 s) and ascent time (208 s) by the tangent of 90° minus the pitch angle for descents (-20.54°) and ascents (+9.78°), resulting in an approximate horizontal distance of 110 m during a descent and 263 m in an ascent (Siders, unpublished data). These ascent and descent rates observed in the Bay of Fundy are much faster than those reported in Shepard et al. (2006) [44] for the eNA (0.11 - 0.37 m min^-1^). Therefore, given the cell size of our model (2.5 x 2.5 km) and short horizontal distances during ascent and descents, it is likely that many surface-swimming sharks were closely tied to suitable habitat at depth. We acknowledge that prolonged surface swimming would confound this argument since an animal could have been at the surface swimming for an indeterminate period before the sighting took place. The dive records revealed that on average sharks were between 0-2 m of the surface 15 ±10% of the time. Although this may introduce some bias between sharks at the surface and at depth, we assert that the surface sightings used in our models are largely representative of suitable habitat at depth.

### Environmental variables

Environmental variables representing the bathymetry, distance to bathymetric contours, chlorophyll-*a* concentration (Chl-*a*), and sea surface temperature (SST) were chosen for our Maxent analysis. We derived six variables at a 0.05 km^2^ resolution – depth, aspect (slope direction), slope angle (in degrees), and distance to the 0 m (shore), 50 m, 100 m, 150 m, and 200 m contours – from a digital bathymetry grid developed by the United States Geological Survey (USGS- http://pubs.usgs.gov/of/1998/of98-801/bathy/data.htm#ArcInfo). Two additional variables- Chl-*a* (in mg m^-3^) and SST (in °C) – were collated at a 16 km^2^ resolution using the Marine Geospatial Ecology Toolbox [45] from the data products of the Moderate Resolution Imaging Spectroradiometer (MODIS-Aqua– http://oceancolor.gsfc.nasa.gov). The remote sensing variables are limited approximations of phytoplankton abundance (Chl-*a*), the base of the phytoplankton-copepod-basking shark food chain, and relative ocean temperature. We calculated the mean of monthly daytime measures available from July to October during 2002-2011 for MODIS-Aqua and pooled all years by month to produce individual environmental variable sets for each time period in R (2013, R Core Development Team). We recognize that year-to-year variation occurred in environmental variables, which could affect shark distributions on small scales; however we intended to generate the first general model of habitat for BoF basking sharks and thus have pooled all years by month. To ensure that significant portions of our sightings data were not excluded when eliminating data-less cells around landmasses, all variables were resampled to a 6.25 km^2^ resolution using *Nearest-neighbor* in ArcGIS 9.3.1 (ESRI Inc., 2009; Redlands, California, USA). All environmental variables were projected on the North American Datum 1983 UTM 19N grid and clipped according to MODIS-Aqua data acquisition from 2002-2011 [imposing a near-shore exclusion zone, ranging from 4.5 km to 9 km]. 

### Spatial autocorrelation

We recognize that our two presence datasets were sampled under different efforts, both spatially and temporally. The commercial whale-watching operation effort was highly clustered, extending from the eastern shore of Grand Manan (Supplemental Material- Figure S1) and was primarily exerted in August and September. The NEAq effort was assumed to be uniform throughout the study area, based on the group’s survey design, and in comparison to the commercial whale-watch effort. We attempted to correct for spatial autocorrelation as a result of these sampling biases but ultimately relied on a subsampling based on the expected average nearest neighbor distance for each month in our dataset. This was determined in ArcGIS via the *Average Nearest Neighbor* routine. Our attempts at correcting for autocorrelation by decimating presence locations by the autocorrelation as detected in a semivariogram and a Moran’s I-distance plot are summarized in the Material. Neither correction was applicable due to the autocorrelation structure detected in the semivariogram (Figure S2) and the decimation routine based on Moran’s I-distance plot (Figure S3) that reduced our sightings by approximately 97%. The sightings for October were not subsampled due to low sample sizes (n ≤ 5) under all routines. However, the NEAq solely collected the sightings in this month and, thus, we assume they were collected in a relatively uniform manner. 

### Species distribution model

#### Maximum entropy modeling

Maxent (version 3.3.3k – see http://www.cs.princeton.edu/~schapire/maxent/) is a machine learning species distribution modeling technique that seeks the probability distribution that maximizes entropy (i.e. closest to uniform) subject to constraints [32]; in our case to estimate basking shark habitat in the BoF in July, August, September, and October [32,33]. Each Maxent estimation was informed using the presence-only sighting data and environmental variables. Environmental covariates are transformed into a set of “features” [32] and we utilized the feature suite available (linear, quadratic, product, threshold, and hinge) [46]. To estimate habitat, Maxent assumes a theoretical uniform distribution then iteratively adjusts the weight of constraints (derived from the empirical mean of environmental variables over the set of species occurrence locations) so that the average probability of sample points is maximized, expressed as training gain. The probability distribution of a species is estimated over the habitat area so each cell holds a probability of presence or relative suitability for the species [47]. We chose to use the cumulative output scaled from 0 to 100, which reports percent relative suitability of a grid cell by calculating the probability of that cell and all cells with equal or lower probability [32]. Maxent prevents overfitting by utilizing a regularization technique to smooth models [46]. We chose unique regularization values (β) for each month following the AICc procedure outlined in Warren & Seifert (2011) [48], which we will summarize here. For each month, models were executed as described above but using β = 1, 3, 5, 7, 9, 11, 13, 15, 17. The suitability scores from these estimations were standardized in order to sum to 1 across the geographic space. The likelihood of a single model’s data was obtained by taking the product of the standardized scores of cells containing presence points. The number of parameters for each estimation was obtained from the lambda file produced during the Maxent routine. AICc was then calculated for each month using sample sizes for each time period and for the suite of β values within each month. The regularization value of the model with the lowest AICc score was used in all subsequent Maxent estimations. All models were performed using a maximum of 100 replicates and a maximum of 10000 background points, which allows Maxent to estimate variance. 

#### Threshold suitability indices

Following previous maximum entropy distribution models [49], we chose the maximum training sensitivity and specificity threshold values. For each time period, unique maximum training sensitivity and specificity thresholds were determined based on Receiving Operator Characteristic (ROC) curves (see Model performance evaluation). 

### Model performance evaluation

#### Metrics of model performance

The most often reported measure of Maxent outputs is the threshold-independent assessment using the Area Under the Curve (AUC) metric of the ROC curve [50]. The ROC curve plots sensitivity values (true positives) against 1 minus specificity (false positive) values [32]. AUC values designate the probability that positive and negative instances are correctly classified. We evaluated AUC values following Phillips et al. (2006): <0.5 (worse than random performance), 0.5-0.7 (no discrimination), >0.7 (better than random model performance), and 1 (perfect discrimination). 

We assessed variable importance using the percent contribution metric [32], which describes the relative contribution to each model. We also assessed permutation importance or the importance of the original background values to the predicted distribution [32]. A high permutation importance indicates that changes to the original data contained within the environmental variable have a strong negative affect on the probability distribution. Finally, we used a jackknife analysis on training data to determine which variables contain the most useful information about the predicted distribution individually, as well as those that [46]. 

#### Model-fitting comparison

Maxent provides three methods to assess model fit: cross-validation, bootstrapping, and subsampling. The number of sightings available in our database in July, August, and September allowed us to perform empirical comparisons of these model-fitting techniques. We performed model runs for those time periods where these techniques were estimated to be more appropriate (July, August, and September) as well as in the October time period to compare a small dataset using all available variables. The random test percentage, used in subsampling, was set to 25% test and 75% training [51].

## Results

### Regularization selection

The AICc model selection procedure selected models with different regularization values for each month; July: β = 5; August: β = 7; September: β = 3; October: β = 7. AICc did not follow any discernible pattern for any of the monthly Maxent models as regularization values were increased from 1 - 17. 

### Model performance

#### Model-fitting comparison

We evaluated the three model-fitting approaches and found that bootstrapping had the lowest false-positive rate as assessed by AUC values, followed by cross-validation, and then by subsampling (Table 1). However, bootstrapping and subsampling produced standard deviation of AUC values 75% lower than cross-validation. The Maxent cumulative-based habitat suitability maps were similar for all models, but the relative suitability standard deviation was lowest in the cross-validation (average across grid cells and models, 0.57), followed by subsampling (1.82), and then bootstrapping (2.89). Given this, we used the cross-validation model-fitting technique. Although we incurred more variation in AUC values using cross-validation (while mean AUC values remain high), we reasoned that lower variance in habitat suitability maps is more importance for estimating areas of critical habitat with more confidence. 

**Table 1:** Summary of the Maximum Entropy models comparing model-fitting routines: cross-validation, bootstrapping, and subsampling.

**Model-fitting**	**Model metrics**	**July**	**August**	**September**	**October**
Cross-validation	AUC ± SD	0.796 ± 0.233	0.926 ± 0.078	0.869 ± 0.171	0.892 ± 0.092
Subsampling	AUC ± SD	0.744 ± 0.089	0.925 ± 0.014	0.865 ± 0.044	0.888 ± 0.047
Bootstrapping	AUC ± SD	0.846 ± 0.071	0.938 ± 0.007	0.919 ± 0.014	0.989 ± 0.033
All routines	Top-ranked PC	Shore	Chl-*a*	Chl-*a*	200 m
	Top-ranked PI	SST*	Chl-*a*	Shore	200 m
	Isolation/Omitted	Shore	Chl-*a*	Chl-a/ Shore	200 m

All models were performed using depth, aspect, slope, distance to shore, distance to 50 m contour, distance to 100 m contour, distance to 150 m contour, distance to 200 m contour, mean chlorophyll *a* (Chl-*a*), and mean sea surface temperature. The top-ranked percent contribution (PC), permutation importance (PI), jackknife tests for variable importance of variables in isolation and variable importance when variables are omitted (Isolation/Omitted) are shown.

* Top-ranked PI was 150 m for the bootstrapping routine in the July model

### Distribution shifts

All Maxent estimations had mean AUC values greater than 0.796, with the August model having the highest, followed by October, September, and then July (Table 2). The variable with the highest percent contribution was split between models: distance to shore in July, chlorophyll-*a* in August and September, and distance to the 200 m contour in October (Table 2). The highest permutation importance was sea surface temperature in July, distance to shore for August and September, but distance to the 200 m contour in October (Table 2). 

**Table 2:** Summary of Maxent model for basking shark distributions in the Bay of Fundy results using crossvalidation.

**Model metrics**	**July**	**August**	**September**	**October**
N	22	110	51	19
ANN	4560 m	1844 m	2920 m	--
Top-ranked PC	Shore (62.2%)	Chl-*a* (58%)	Chl-*a* (50.8%)	200 m (77.2%)
2^nd^ ranked PC	150 m (17.0%)	150 m (12.6%)	Shore (18.6%)	100 m (22.5%)
3^rd^ ranked PC	SST (15.3%)	Shore (9.2%)	200 m (13.2%)	Slope (0.2%)
Top-ranked PI	SST (38.9%)	Shore (39.1%)	Shore (58%)	200m (79.9%)
1^st^ Isolation/Omitted	Shore	Chl-*a*	Chl-*a*/Shore	200 m/ 200 m
AUC ± SD	0.796 ± 0.233	0.926 ± 0.078	0.869 ± 0.171	0.892 ± 0.092

The numbers of sightings (N) used in the modeling routine as well as the expected average nearest neighbor distance (ANN) used to select sighting locations. The top-, 2^nd^-, and 3^rd^-ranked percent contributions (PC), top-ranked permutation importance (PI), top-ranked jackknife tests for variable importance of variables in isolation and variable importance when variables are omitted (Isolation/Omitted) are shown.

**Figure 2:**
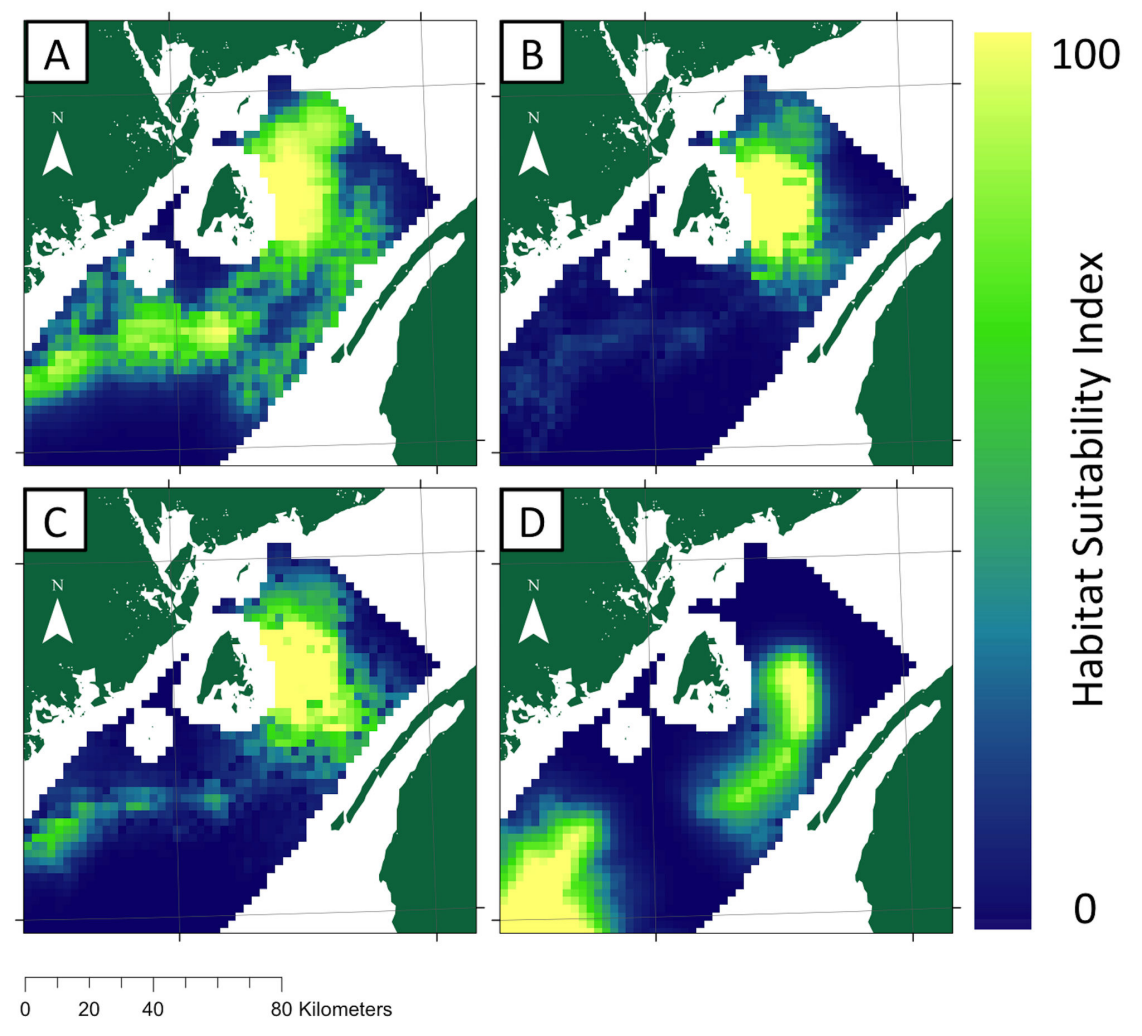
Maps of relative habitat suitability of basking sharks in the lower Bay of Fundy. Maxent distribution models used environmental predictors were chlorophyll-a, sea surface temperature, depth, aspect, slope, and distance to the 0, 50, 100, 150, 200 m contours for (A) July, (B) August, (C) September, and (D) October. Warmer colors indicate higher suitability.

Overall, habitat of the highest suitability shifted minimally between August and September (>0.84 between each models, Brownian distance correlation coefficient (BDCC), [52]) (Figure 2). July was the next most similar distribution (>0.44 compared to August and September, BDCC) but predicted a larger extent of suitable habitat (Table 2). All three models predicted the most suitable habitat in the northeast Grand Manan Basin, straddling the 150 m contour and extending southward to the 200 m contour. The main difference between these distributions was the extent of suitable habitat predicted around Murr Ledges (see Figure 1a for location); models for July (Figure 2a) predicted this as highly suitable habitat while models for August (Figure 2b) and September (Figure 2c) did not. Models for July, August, and September all predicted suitable habitat in Jones Ground (see Figure 1a for location) in the northeastern margin of the GoM (Figure 2a-c), though considerably less was predicted in August. Habitat suitability differed in October (<0.036 compared to all other time periods, BDCC) revealing the most suitable habitat near Jones Ground as well as the southern portion of the Grand Manan Basin near the 200 m contour (Figure 2d). 

Variable response curves driving the shifts between time periods varied considerably but several trends emerged (Table 2). Throughout July to September, the percent contribution from distance to the shore was in the top three but the model responses varied from month to month (1^st^ ranked - July, < 15 km; 3^rd^ ranked - August, < 23 km; 2^nd^ ranked - September, < 24 km) (Figure S4). In July and August, distance to 150 m contributed the second most to these models (selecting for distances < 3 km and < 7 km from the contour, respectively) (Figure S5). Chlorophyll-*a* was the highest contributor to the August and September models and responded to concentrations 2.25 - 4 mg m^-3^ and > 2 mg m^-3^ (Figure S6). In September and October, distance to the 200 m contour was the third and first ranked contributor (selecting for distances < 20 km and < 7 km, respectively) (Figure S7). In July, sea-surface temperature was the third ranked contributor to the model and the variable response was to temperatures 13.2 - 13.8°C (Figure S8). Distance to the 100 m contour was the second ranked contributor to the October model (selecting for distances > 7 km from the contour) (Figure S9). 

### Threshold habitat values

**Figure 3:**
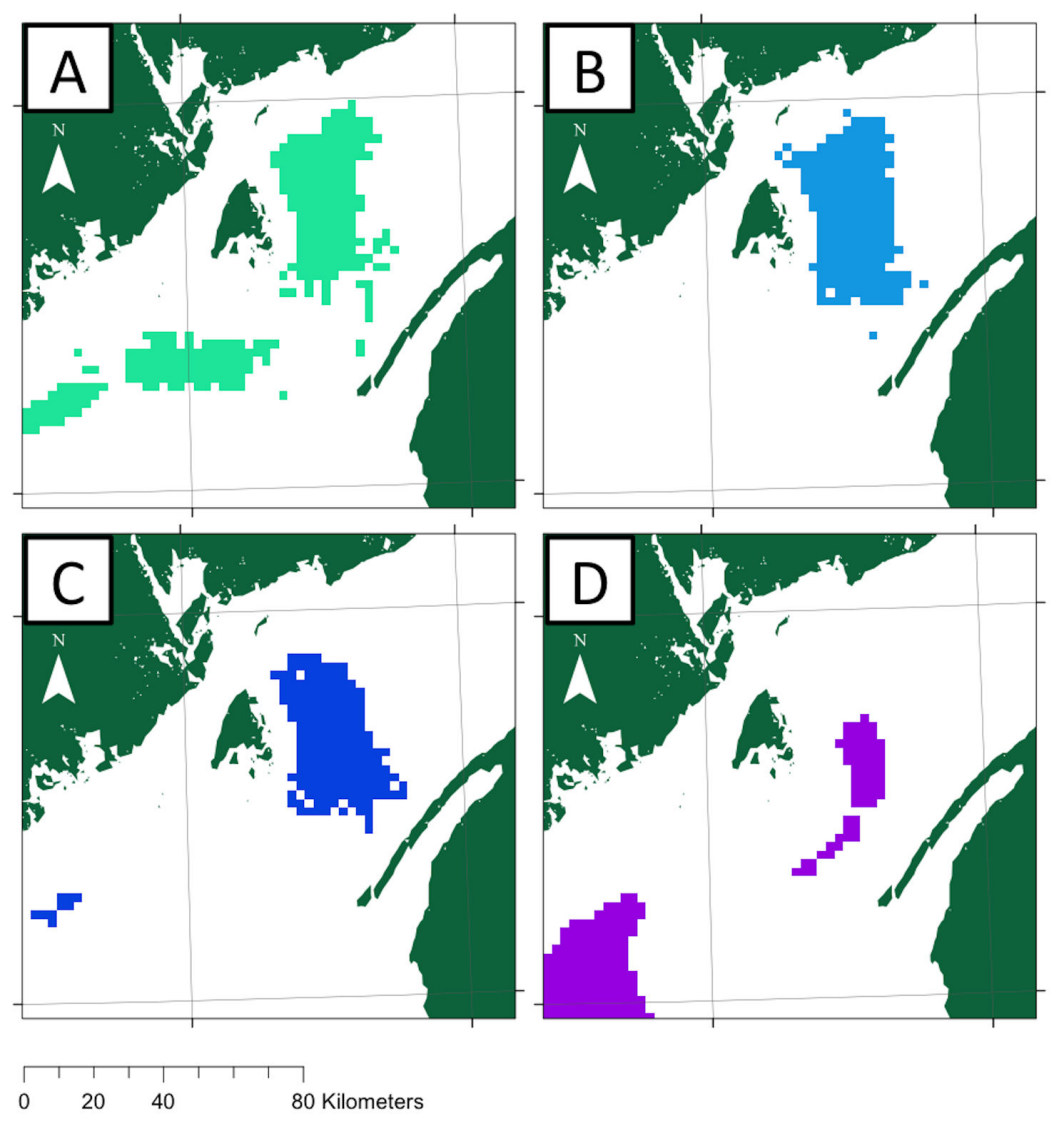
Polygons of threshold defined suitable basking shark habitat. The maximum sensitivity and specificity threshold was used to distinguish suitable (colored) and unsuitable habitat (white) from Maxent distribution model predictions for July (A), August (B), September (C), October (D).

Based on the ROC curve for each model, the maximum sensitivity and specificity cumulative suitability score threshold varied (July, 55,63; August, 14.22; September, 30.20; October, 50.33) (Table 3). These thresholds resulted in different amounts of the habitat that were above the threshold and thus likely to be highly suitable basking shark habitat (July, 25.64%; August, 16.89%; September, 14.96%; October, 15.75%) (Table 3). The locations of this habitat occurred throughout the study area in July, almost entirely in the Bay of Fundy in August and September, then shifted in October so that a majority of habitat was in the Gulf of Maine (Figure 3). 

**Table 3:** Summary of threshold habitat suitability evaluations.

**Approach**	**July**	**August**	**September**	**October**
Threshold value	55.63	14.22	30.20	50.33
Suitable area (km^2^)	1933	1273	1128	1187
Percent suitable habitat	25.64%	16.89%	14.96%	15.75%

The threshold value, suitable area in square kilometers, and percent suitable habitat of total habitat are given.

## Discussion

Basking sharks in the western North Atlantic inhabit coastal waters [19] but this is the first study to model their spatial distribution. All of our Maxent estimations performed well, as assessed by AUC values, despite different model-fitting techniques, different combinations of variables, and variables of different resolutions. We conclude from these results that the maximum entropy approach is appropriate for modeling habitat suitability of basking sharks. Despite variation in the predicted habitat location, a majority of habitat occurs within the BoF from July-September. The localization of this habitat presents an opportunity to protect this species of S*pecial Concern* in this area [7]. 

### Model performance

The results of our Maxent models reveal the consequences of using different model-fitting techniques of bootstrapping, subsampling, and cross-validation. We chose to use cross-validation because our primary focus was assessing spatial differences in basking shark distributions, not overall model performance. We show that when using other model-fitting techniques within Maxent with large presence-only data sets, a trade-off between model performance and variation in habitat suitability predictions may occur. The trade-off is relatively simple when models perform well. However, as model performance deteriorates, sacrifices in habitat suitability confidence may be necessary to maintain adequate model function. As we demonstrated, using the bootstrapping model-fitting technique improved model performance both in mean and standard deviation of AUC values but at the sacrifice of increasing uncertainty in habitat suitability maps. Importantly, the change from cross-validation to either subsampling or bootstrapping did not change variable importance.

### Implications of spatial heterogeneity and temporal shifts

Models for July-October denoted variation in the distribution of basking sharks (Figure 2 & 3) and these distributional changes are likely driven by changes in the distribution and relative concentration of their main prey. Basking sharks in the BoF would encounter the densest swarms of C5 copepods in the Grand Manan Basin during August [26,28,53]. The rotational transit time of the seasonal BoF Gyre increases the basal residence time of zooplankton within the BoF [54] and increases the concentration of C5 copepods. If, like NA right whales, basking sharks feed on C5s only during summer, then it would be adaptive for them to be tied closely to their prey especially during times when it is more concentrated (Figure 2b). 

Owen (1984) also observed evidence for habitat shifts by documenting basking shark distributions in the GoM changing from shallow water to deep water in September. Our model predicted an earlier shift to deep-water habitats in the BoF (August) that may reflect differences in the prey field between these habitats. Owen (1984) documented copepod swarms in shallow (<80 m) waters of the GoM throughout the summer and observed surface feeding sharks primarily along the western edge of Jordan Basin from June to August. These patches were likely *C. finmarchicus* C4 and C5, presumably feeding on phytoplankton prior to the C5 diapause [55,56]. Habitat shifts have also been documented in the eNA, where basking sharks moved from the neritic zone, consuming primarily shallow-water *Calanus helgolandicus*, to the continental edge where they consumed primarily deep-water *C. finmarchicus* [13]. 

Interestingly, the narrow bands of suitable habitat along the Murr Ledges in the July suitability map overlap with the outflow of the cyclonic Bay of Fundy Gyre [20] (Figure 2a and see Figure S10). This suggests basking sharks may follow the advection of prey via these fast currents when high concentrations of prey are not as available as in August and September. Similarly in the eNA, basking sharks have exhibited area-restrictive foraging by closely following thermal and tidal fronts as well as prey density differences [57,58]. 

The appearance of suitable habitat further south in September (Figure 2d) likely represents the onset of southerly movement of basking sharks out of the BoF. Limited observations of basking sharks moving from the western margins of the GoM to the northwestern margin of Georges Bank [south of the GoM] (Owen, 1984) tentatively support this. Pop-up satellite archival tag deployments in the BoF (Westgate A.J., unpublished data) have shown that basking sharks typically leave BoF/GoM waters between mid-October and mid-November, quickly moving into much deeper water off the shelf break. Three hypotheses have been suggested for a southward distributional shift: 1) basking sharks are following the movements (both vertically and horizontally) of the most energetically favorable prey [12,59–61] (Parker & Boeseman, 1954; Owen, 1984; Sims, 1999; Sims et al., 2005); 2) basking sharks are leaving prey patches in the BoF as abundances declines [28]; or 3) ectothermic basking sharks must maintain body temperatures above some threshold [12,15] and thus move to where ocean temperatures are suitable [57].

Southerly movement of the basking shark distribution, as predicted in our October model (Figure 2d), does not exclusively support any of these hypotheses. As the environmental variables used in this study, Chl-*a* and SST, are limited to describing surface changes and cannot assess C5 *C. finmarchicus* dynamics or thermal stratification, we can only speculate on the mechanism behind distribution shifts. Owen (1984) proposed that basking sharks in the wNA may follow warm surface water as it undergoes vertical mixing because animals are thermally constrained, allowing October (or later) access to deeper-water habitats, thus eliminating basking sharks from surface sighting records. The shift to deep-water habitats in the October distribution in this study likely reflects such a movement as the model shifted from 180 m in depth in July-September to > 200 m in October-December. Additionally, response to the distance to the 200 m contour decreased from 20 km from the contour in September to less than 7 km in October. However, SST did not strongly influence the October distribution which implies that temperature is less important than prey requirements or that SST does not capture the thermal environment basking sharks are responding to [13,58,61]. Additionally, the breakdown of thermal stratification weakens the cyclonic Bay of Fundy Gyre [20], and may cause C5 ejection into the GoM, which basking sharks may follow. Since we do not understand the complexities of phytoplankton, copepod and basking shark trophodynamics in this region these findings are difficult to evaluate with any certainty.

### Threshold habitat values

The model predictions have shown that sharks regularly use select areas within the BoF. These select areas may offer opportunities for management strategies, such as shifting traffic patterns and creating protection zones for basking sharks in the northern Grand Manan Basin [19,62]. Habitat suitability as defined by the maximum sensitivity and specificity threshold significantly reduced predicted habitat area, ranging from 14.86 to 25.64% of the total habitat. This has broad implications for conservation measures, as protection of a fraction of the total habitat would translate to protection for a disproportionately greater number of sharks. It is important to note, however, that the threshold index is derived from the modeling process and associated ecological inferences, and should be validated through an independent long-term dataset, such as a decade-long aerial survey series, or via satellite telemetry tagging on a significant number of sharks. 

## Conclusion

We have developed the first maximum entropy model for basking sharks and made predictions of their distribution in the BoF. Suitable habitat was shown to vary between July-October over 2002-2011, indicating heterogeneous habitat utilization. We hypothesized these distributional shifts are related to prey dynamics but further understanding of the ecological drivers is necessary to elucidate the specific mechanisms of basking shark movements in the western North Atlantic. Also, we have compared model-fitting routines and reasoned that their choice must be weighed against study objectives. Defining the spatial distribution of basking sharks, as we have done, represents a critical step in future efforts towards conserving and understanding this large but understudied species, especially in data deficient areas such as Atlantic Canada. 

## Supporting Information

Figure S1 **Map for July-October of the distribution of sampling effort throughout the study area.**
(DOCX)

Figure S2 **Semi-variograms generated from sighting locations and environmental variables showing the spatial autocorrelation in the dataset used for Maxent estimations.**
(DOCX)

Figure S3 **Moran’s I of sightings locations and environmental variables as a function of distance used in an attempt to decimate sampling locations in order to correct for spatial autocorrelation.**
(DOCX)

Figure S4 **Model responses to distance to shore for July-September against a histogram of distance to shore in the study area.**
(DOCX)

Figure S5 **Model responses to distance to the 150 m contour for July and August against a histogram of distance to the 150 m contour in the study area.**
(DOCX)

Figure S6 **Model responses to chlorophyll-*a* for August and September against a histogram of chlorophyll-*a* in August and September in the study area.**
(DOCX)

Figure S7 **Model responses to distance to 200 m contour for September and October against a histogram of distance to 200 m contour in the study area.**
(DOCX)

Figure S8 **Model responses to sea surface temperature for July against a histogram of sea surface temperature in July in the study area.**
(DOCX)

Figure S9 **Model responses to distance to the 100 m contour for October against a histogram of distance to the 100 m contour in the study area.**
(DOCX)

Figure S10 **Map of major currents affecting the Bay of Fundy Gyre circulation.**
(DOCX)
