# Supplementary material for: Seasonal Variation in the Spatial Distribution of Basking Sharks (Cetorhinus maximus) in the Lower Bay of Fundy, Canada
Source: PLoS One. 2013 Dec 4;8(12):e82074. doi: 10.1371/journal.pone.0082074 (PMC3852988; doi:10.1371/journal.pone.0082074)
Supplement: Figure S1 — Map for July-October of the distribution of sampling effort throughout the study area. (DOCX) [file pone.0082074.s001.docx]

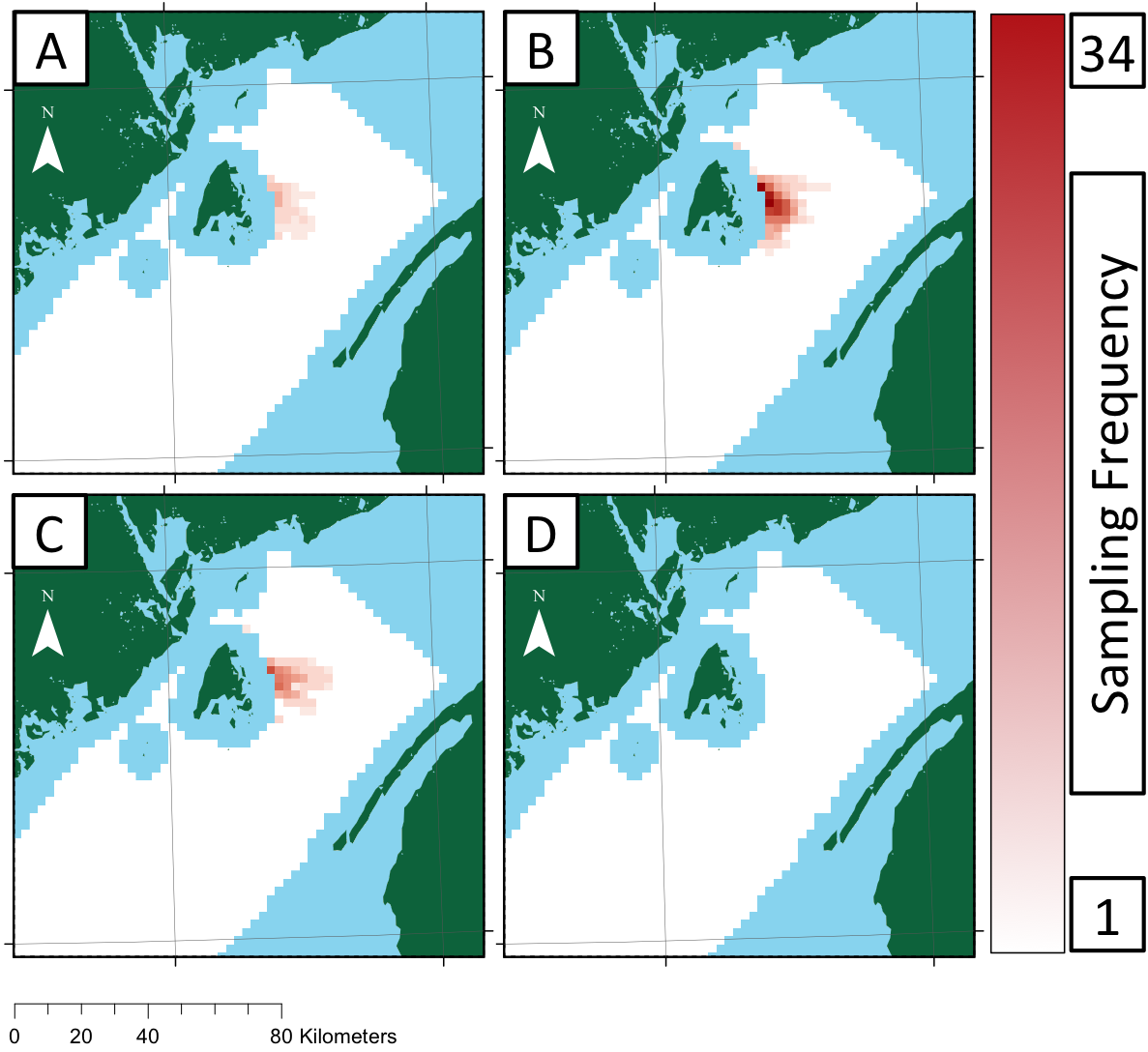


Figure S1: Maps of the sampling effort in the Bay of Fundy for basking sharks in A) July, B) August, C) September, D) October, for a commercial whale-watching operation. Deeper reds indicate more visits than lighter areas. The uniform sampling effort of the second set of sighting locations from the New England Aquarium was as a visit of 1 to every cell thus setting the baseline number of visits. This maps were used as “Bias Files” in the Maxent estimations for each month.
