# Supplementary material for: Seasonal Variation in the Spatial Distribution of Basking Sharks (Cetorhinus maximus) in the Lower Bay of Fundy, Canada
Source: PLoS One. 2013 Dec 4;8(12):e82074. doi: 10.1371/journal.pone.0082074 (PMC3852988; doi:10.1371/journal.pone.0082074)
Supplement: Figure S2 — Semi-variograms generated from sighting locations and environmental variables showing the spatial autocorrelation in the dataset used for Maxent estimations. (DOCX) [file pone.0082074.s002.docx]

Figure S2: Semi-variograms of the habitat index for each sighting location. The left panel is the raw semivariance for each sampling point in July from our basking shark sighting location dataset. The right panel is the binned semivariance of those points. The bimodal nature of this plot limits the ability to discern a cutoff distance where autocorrelation dissipates.
