# Supplementary material for: Seasonal Variation in the Spatial Distribution of Basking Sharks (Cetorhinus maximus) in the Lower Bay of Fundy, Canada
Source: PLoS One. 2013 Dec 4;8(12):e82074. doi: 10.1371/journal.pone.0082074 (PMC3852988; doi:10.1371/journal.pone.0082074)
Supplement: Figure S3 — Moran’s I of sightings locations and environmental variables as a function of distance used in an attempt to decimate sampling locations in order to correct for spatial autocorrelation. (DOCX) [file pone.0082074.s003.docx]

Figure S3: Moran’s I-distance plot detailing the mean Moran’s I values for sighting locations within each distance bin. Red points indicate significantly different positive Moran’s I values than those expected for that distance bin. From this plot we were able to determine that the autocorrelation structure dissipates four grid cells away from a given sighting location. However, decimating sampling locations using this distance resulted in up to a 97% decrease in our sample sizes.
