# Supplementary material for: Seasonal Variation in the Spatial Distribution of Basking Sharks (Cetorhinus maximus) in the Lower Bay of Fundy, Canada
Source: PLoS One. 2013 Dec 4;8(12):e82074. doi: 10.1371/journal.pone.0082074 (PMC3852988; doi:10.1371/journal.pone.0082074)
Supplement: Figure S4 — Model responses to distance to shore for July-September against a histogram of distance to shore in the study area. (DOCX) [file pone.0082074.s004.docx]

Figure S4: Histogram of the distance to the shore values in the raw environmental layer plotted against the Maxent model response for July (aquamarine), August (light blue), and September (blue), where distance to shore was one of the top three variables contributing to the model.
