# Supplementary material for: Seasonal Variation in the Spatial Distribution of Basking Sharks (Cetorhinus maximus) in the Lower Bay of Fundy, Canada
Source: PLoS One. 2013 Dec 4;8(12):e82074. doi: 10.1371/journal.pone.0082074 (PMC3852988; doi:10.1371/journal.pone.0082074)
Supplement: Figure S6 — Model responses to chlorophyll-a for August and September against a histogram of chlorophyll-a in August and September in the study area. (DOCX) [file pone.0082074.s006.docx]

Figure S6: Histogram of the chlorophyll-*a* values for August (dark gray) and September (light gray) in the raw environmental layer plotted against the Maxent model response for August (light blue) and September (blue), where chlorophyll-*a* was the top variable contributing to the model.
