# Supplementary material for: Seasonal Variation in the Spatial Distribution of Basking Sharks (Cetorhinus maximus) in the Lower Bay of Fundy, Canada
Source: PLoS One. 2013 Dec 4;8(12):e82074. doi: 10.1371/journal.pone.0082074 (PMC3852988; doi:10.1371/journal.pone.0082074)
Supplement: Figure S7 — Model responses to distance to 200 m contour for September and October against a histogram of distance to 200 m contour in the study area. (DOCX) [file pone.0082074.s007.docx]

Figure S7: Histogram of the distance to the 200 m contour values in the raw environmental layer plotted against the Maxent model response for September (blue) and October (violet), where distance to 200 m contour was one of the top three variables contributing to the model.
