# Supplementary material for: Seasonal Variation in the Spatial Distribution of Basking Sharks (Cetorhinus maximus) in the Lower Bay of Fundy, Canada
Source: PLoS One. 2013 Dec 4;8(12):e82074. doi: 10.1371/journal.pone.0082074 (PMC3852988; doi:10.1371/journal.pone.0082074)
Supplement: Figure S8 — Model responses to sea surface temperature for July against a histogram of sea surface temperature in July in the study area. (DOCX) [file pone.0082074.s008.docx]

Figure S8: Histogram of the sea surface temperature values for July in the raw environmental layer plotted against the Maxent model response for July, where sea surface temperature was the third ranked variable contributing to the model.
