# Supplementary material for: Seasonal Variation in the Spatial Distribution of Basking Sharks (Cetorhinus maximus) in the Lower Bay of Fundy, Canada
Source: PLoS One. 2013 Dec 4;8(12):e82074. doi: 10.1371/journal.pone.0082074 (PMC3852988; doi:10.1371/journal.pone.0082074)
Supplement: Figure S9 — Model responses to distance to the 100 m contour for October against a histogram of distance to the 100 m contour in the study area. (DOCX) [file pone.0082074.s009.docx]

Figure S9: Histogram of the distance to the 100 m contour values for October in the raw environmental layer plotted against the Maxent model response for October, where distance to the 100 m contour was second ranked variable contributing to the model.
