# Supplementary material for: Seasonal Variation in the Spatial Distribution of Basking Sharks (Cetorhinus maximus) in the Lower Bay of Fundy, Canada
Source: PLoS One. 2013 Dec 4;8(12):e82074. doi: 10.1371/journal.pone.0082074 (PMC3852988; doi:10.1371/journal.pone.0082074)
Supplement: Figure S10 — Map of major currents affecting the Bay of Fundy Gyre circulation. (DOCX) [file pone.0082074.s010.docx]

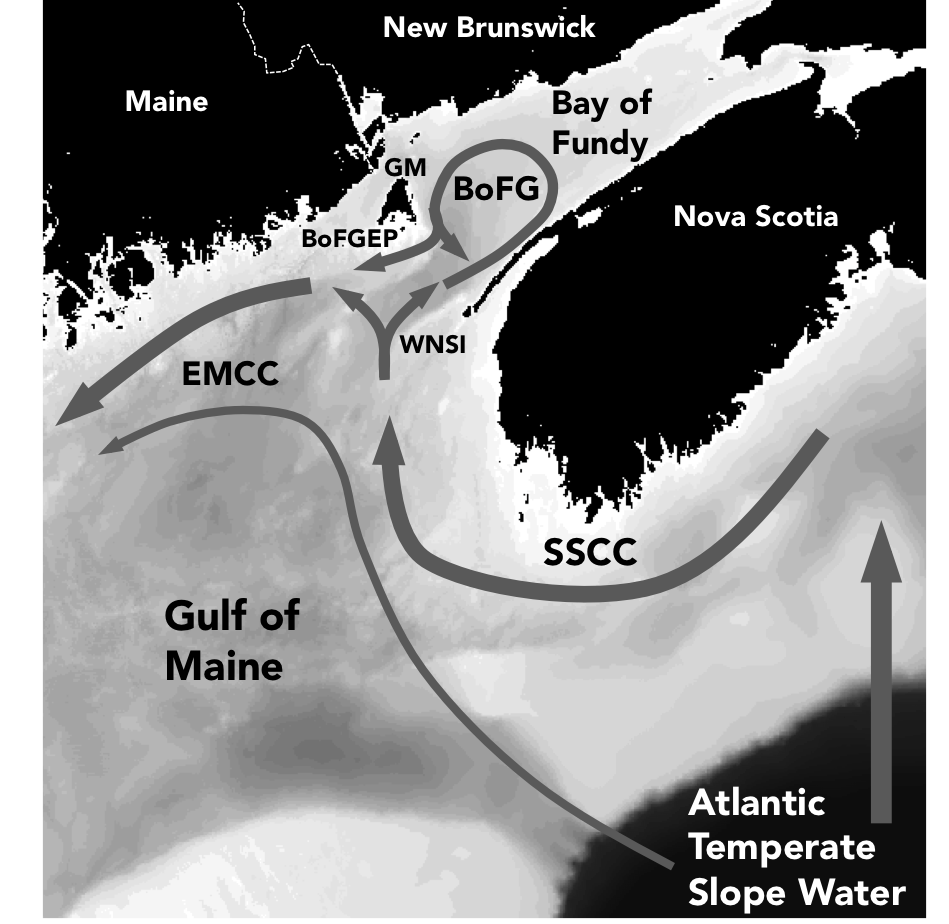


Figure S10: Map of the major current boundaries in the eastern Gulf of Maine, western Scotian Shelf, and Bay of Fundy. The Bay of Fundy Gyre (BoFG) is located on the eastern side of Grand Manan (GM) and developed by inflow through the Western Nova Scotian Inflow (WNSI) and outflow through the Bay of Fundy Gyre Exit Pathway (BoFGEP). The advection of Atlantic Temperate Slope Water contributes warmer water to the relatively cooled Scotian Shelf Coastal Current (SSCC), which subsequently forms a majority of the BoFG flow, and the Eastern Maine Coastal Current (EMCC). Naming convention follows Aretxabaleta et al. (2008).
